# Health literacy in gastrointestinal diseases: a comparative analysis between patients with liver cirrhosis, inflammatory bowel disease and gastrointestinal cancer

**DOI:** 10.1038/s41598-022-25699-w

**Published:** 2022-12-06

**Authors:** Leonard Kaps, Lea Omogbehin, Katharina Hildebrand, Simon J. Gairing, Eva M. Schleicher, Markus Moehler, Fareed Rahman, Jörn M. Schattenberg, Marcus-Alexander Wörns, Peter R. Galle, Christian Labenz

**Affiliations:** 1 Department of Internal Medicine I, University Medical Center of the Johannes Gutenberg-University, Langenbeckstrasse 1, 55131 Mainz, Germany; 2 Cirrhosis Center Mainz (CCM), University Medical Center of the Johannes Gutenberg-University, Mainz, Germany; 3 Department of Gastroenterology, Hematology, Oncology and Endocrinology, Klinikum Dortmund, Dortmund, Germany

**Keywords:** Gastroenterology, Hepatology

## Abstract

Currently, there are only few data on health literacy in patients with chronic gastrointestinal diseases such as gastrointestinal cancer, inflammatory bowel disease (IBD) and, in particular, liver cirrhosis available. Moreover, head-to-head comparisons between patients with these different diseases are lacking. In this study, 379 patients were enrolled. Of these, 102 patients had gastrointestinal cancer, 86 had IBD, and 191 had cirrhosis. Health literacy was quantified using the Health Literacy Questionnaire (HLQ) developed by Osborne et al. (Swinburne University, Australia) and was compared between these three groups. Patients with cancer had the best health literacy across all nine subscales of the HLQ, while patients with cirrhosis had the poorest. In detail, patients with cirrhosis had significantly poorer health literacy than patients with cancer or IBD in subscales such as “feeling understood and supported by healthcare providers”, “having sufficient information to manage my health”, “appraisal of health information”, “ability to actively engage with healthcare providers” or “understanding health information well enough to know what to do” (p < 0.05 for cirrhosis versus IBD or cancer, respectively). In conclusion, health literacy differs remarkably between patients with chronic gastrointestinal diseases such as cirrhosis, IBD or gastrointestinal cancers.

## Introduction

Modern healthcare systems tend to be increasingly complex, posing a challenge for patients with chronic diseases to manage their diseases for optimal healthcare^1,2^. Health literacy is a concept that covers key determinants of patient’s ability to obtain and use health information to make health decisions, access care in the community, structure self-care, promote provider-patient interactions, and navigate the health care system^3,4^. There is mounting evidence that poor health literacy is associated with increased hospitalization, health system costs, and mortality, through insufficient adherence to healthcare services, medical check-ups, medication errors, and finally with difficulties to communicate with healthcare professionals and poor knowledge about disease processes^5–9^. Thus, poor health illiteracy may have a particular impact on patients with chronic diseases, who need ongoing support from their healthcare providers^10,11^.

Some of the most common and relevant gastrointestinal diseases in the Western world are liver cirrhosis, inflammatory bowel diseases (IBD) and gastrointestinal cancers. Worldwide, about 122 million patients suffer from liver cirrhosis and 6.8 million from IBD^12,13^. The estimated incidence of gastrointestinal cancers is as high as 4.8 million new cases per year, and they account for 26% of the global cancer incidence^14^. Given the complexity in the management of these diseases, not only for physicians and caregivers, but also for patients, a sufficient level of health literacy seems mandatory to manage one’s own disease. While health literacy has been studied in patients with various cancers^15,16^, data on health literacy in patients with IBD and liver cirrhosis are still scarce^17,18^. Furthermore, especially data on head-to-head comparisons of health literacy in patients living with these diseases are currently lacking. However, it should be emphasized that data on this topic are critically needed to identify high-risk groups for poorer health literacy to enable more intensive support from the healthcare care system to improve prognosis and overcome disparities. Therefore, we aimed to investigate and compare health literacy of patients with gastrointestinal cancer, IBD, and liver cirrhosis using the validated Health Literacy Questionnaire (HLQ) developed by Osborne et al.^19^ (Swinburne University, Australia).

## Results

### Cohort description

In total, 379 patients were prospectively enrolled between January 2019 and April 2022. Of these, 102 patients were diagnosed with cancer, 86 with IBD and 191 patients with liver cirrhosis. Patients with cancer had either esophageal cancer (27.5%), colon cancer (7.8%), pancreatic cancer (16.7%), cholangiocarcinoma (12.7%), rectal cancer (16.7%), gastric cancer (8.8%) or other cancer entities (9.8%). The frequency of Crohn’s disease and ulcerative colitis was balanced among patients with IBD with a frequency of 48.9% and 47.7%, respectively. Only four patients had indeterminate colitis. The predominant etiology of underlying liver disease in patients with liver cirrhosis was chronic alcohol consumption (60.2%), followed by non-alcoholic fatty liver disease (14.7%) and viral hepatitis (8.9%). Most patients with liver cirrhosis were in a decompensated stage of their disease (Child–Pugh A/B/C: 24.6%/49.7%/25.7%). Patients with liver cirrhosis and cancer were older and more frequently male than patients with IBD. The frequency of patients with a university degree was highest in patients with cancer. Additional characteristics of the cohorts are displayed in Table 1 and supplementary tables 1, 2 and 3.

**Table 1:** Demographics and clinical characteristics of the three cohorts.

Variable	Patients with liver cirrhosis n = 191	Patients with cancer n = 102	Patients with IBD n = 86	p-value between groups
Age, y (IQR)	60 (52; 67)^b^	62 (55; 70)^c^	48 (33; 61)^b, c^	** < 0.001**
Male gender, n (%)	117 (61.3)^a^	80 (78.4)^a, c^	42 (48.8)^c^	** < 0.001**
University degree, n (%)	33 (17.3)^a^	36 (35.3)^a^	25 (29.1)	**0.002**
Born outside of Germany, n (%)	25 (13.1)	14 (13.7)	9 (10.5)	ns
**Continent of birth, n (%)**				ns
Europe	187 (97.9)	98 (96.1)	81 (94.2)	
Northern America	0 (0.0)	1 (1.0)	0 (0.0)	
Asia	4 (2.1)	3 (2.9)	3 (3.5)	
Africa	0 (0.0)	0 (0.0)	2 (2.3)	
Speaking German at home, n (%)	179 (93.7)	98 (96.1)	82 (95.3)	ns
**Health literacy questionnaire**				
Scale 1 (IQR)	3.0 (2.75; 3.5)^a, b^	3.0 (3.0; 3.75)^a^	3.25 (3.0; 3.5)^b^	** < 0.001**
Scale 2 (IQR)	3.0 (2.25; 3.0)^a, b^	3.0 (2.75; 3.1)^a^	3.0 (2.75;3.25)^b^	** < 0.001**
Scale 3 (IQR)	2.8 (2.2; 3.0)^a^	3.0 (2.8; 3.2)^a, c^	2.8 (2.6; 3.0)^c^	** < 0.001**
Scale 4 (IQR)	3.2 (2.6; 3.6)^a^	3.4 (3.0; 3.7)^a, c^	3.0 (2.8; 3.6)^c^	** < 0.001**
Scale 5 (IQR)	2.6 (2.0; 3.0)^a, b^	2.8 (2.4; 3.0)^a^	2.8 (2.4; 3.0)^b^	** < 0.001**
Scale 6 (IQR)	3.6 (3.0; 4.0)^a, b^	4.0 (3.4; 4.2)^a^	3.8 (3.2; 4.0)^b^	** < 0.001**
Scale 7 (IQR)	3.5 (3.0; 3.8)^a^	3.7 (3.3; 4.0)^a^	3.5 (3.2; 4.0)	**0.022**
Scale 8 (IQR)	3.4 (3.0; 4.0)	3.6 (3.2; 4.0)	3.6 (3.2; 4.0)	**0.030**
Scale 9 (IQR)	3.4 (3.0; 3.8)^a, b^	3.8 (3.3; 4.0)^a^	3.8 (3.4; 4.0)^b^	** < 0.001**

Significant values are in [bold].

Data are expressed as medians and interquartile ranges or as frequencies and percentages. Comparisons of three groups were performed using an ANOVA followed by Tukey’s multiple comparison test or using chi-squared test.

Scale 1: Feeling understood and supported by healthcare providers, Scale 2: Having sufficient information to manage my health, Scale 3: Actively managing my health, Scale 4: Social support for health, Scale 5: Appraisal of health information, Scale 6: Ability to actively engage with healthcare providers, Scale 7: Navigating the healthcare system, Scale 8: Ability to find good health information, Scale 9: Understanding health information well enough to know what to do.

^a^Indicates significant (p < 0.05) differences of characteristics of patients with liver cirrhosis vs. cancer.

^b^Indicates significant (p < 0.05) differences of characteristics of patients with liver cirrhosis vs. IBD.

^c^Indicates significant (p < 0.05) differences of characteristics of patients with cancer vs. IBD.

### Unadjusted comparison of health literacy between the patient groups

**Figure 1:**
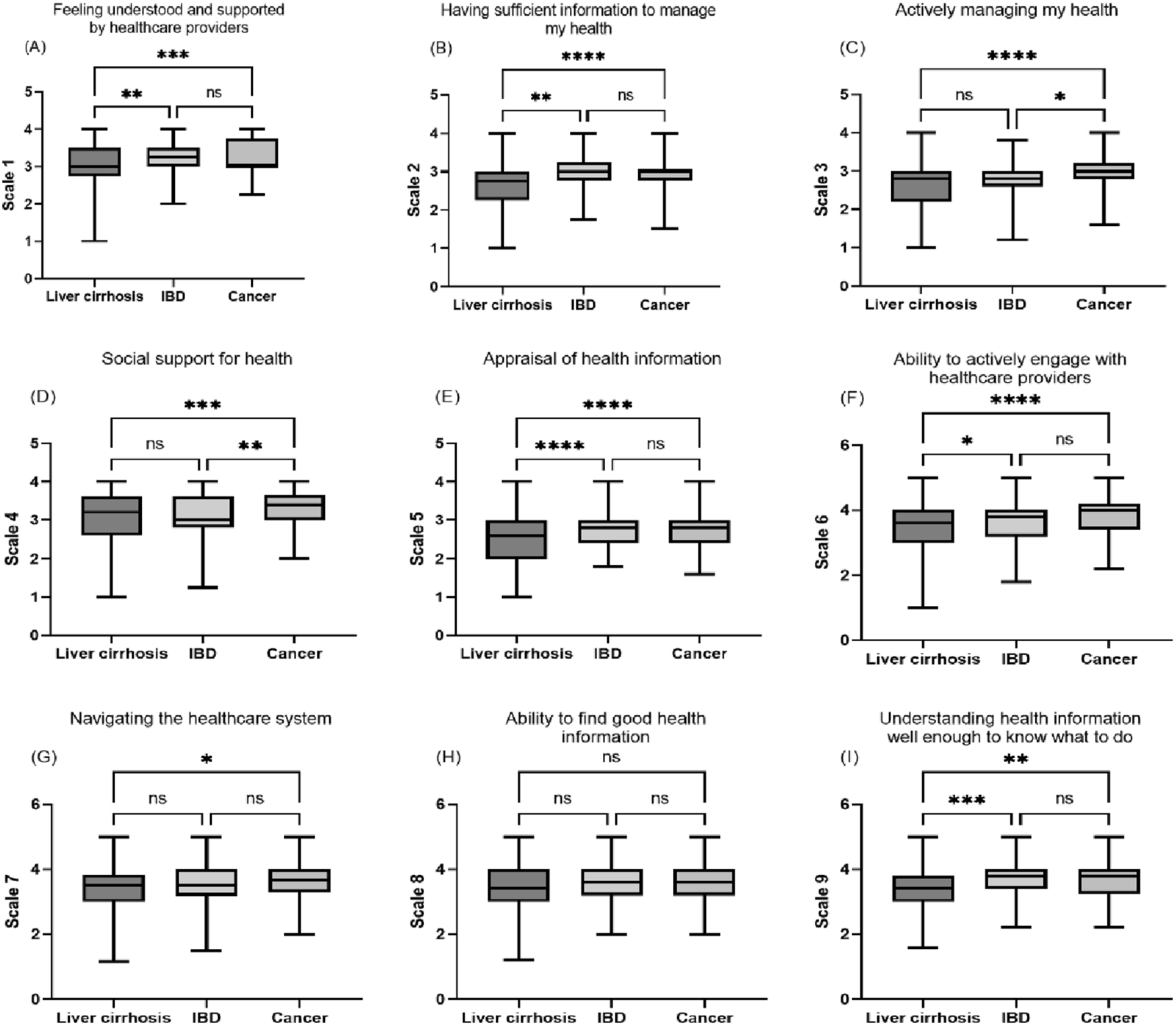
Comparison of the scores in the nine subscales of the HLQ between patients with liver cirrhosis, inflammatory bowel disease (IBD) and cancer. *p < 0.05, ** p < 0.01, *** p < 0.001, **** p < 0.0001, ns = not significant. Comparisons of three groups were performed using an ANOVA followed by Tukey’s multiple comparison test.

First, the scores in the different subscales of the HLQ were compared between patients with cancer, IBD and liver cirrhosis by means of univariable analyses (Fig. 1, Table 1). Here, the scores of patients with liver cirrhosis were significantly lower compared to patients with IBD and cancer in subscales of the HLQ defining important determinants of health literacy such as scale 1 “feeling understood and supported by healthcare providers” (cirrhosis vs. IBD: p < 0.01, cirrhosis vs. cancer: p < 0.001), scale 2 “having sufficient information to manage my health” (cirrhosis vs. IBD: p < 0.01, cirrhosis vs. cancer: p < 0.0001), scale 5 “appraisal of health information” (cirrhosis vs. IBD: p < 0.0001, cirrhosis vs. cancer: p < 0.0001), scale 6 “ability to actively engage with healthcare providers” (cirrhosis vs. IBD: p < 0.05, cirrhosis vs. cancer: p < 0.0001) or scale 9 “understanding health information well enough to know what to do” (cirrhosis vs. IBD: p < 0.001, cirrhosis vs. cancer: p < 0.01). Patients with liver cirrhosis and IBD also had significantly lower scores in scale 3 and 4 than patients with cancer, which define the dimensions “actively managing my health” (cirrhosis vs. cancer: p < 0.0001, IBD vs. cancer: p < 0.05) and “social support for health” (cirrhosis vs. cancer: p < 0.001, IBD vs. cancer: p < 0.01). There was no difference between the three patient groups in terms of the ability to find good health information (scale 8).

### Adjusted comparison of health literacy between the patient groups

To exclude the potential bias caused by differences in age, gender distribution or education (university degree), we conducted various multivariable linear regression analyses for the following comparisons: liver cirrhosis vs. cancer, liver cirrhosis vs. IBD and IBD vs. cancer. All aforementioned variables were subsequently considered in multivariable linear regression models for each subscale of the HLQ. The detailed results of these analyses are displayed in Tables 2, 3 and 4. In brief, liver cirrhosis was associated with poorer scores among all subscales of the HLQ except for scale 8 when compared to patients with cancer (Table 2). In scale 8 (“ability to find good health information”), there was a trend for poorer scores in patients with liver cirrhosis compared to patients with cancer (β = − 0.101, p = 0.089). When comparing patients with liver cirrhosis to patients with IBD in multivariable analyses, patients with liver cirrhosis had significantly poorer scores in scale 1 (“feeling understood and supported by healthcare providers”) (β = − 0.160, p = 0.014), scale 2 (“having sufficient information to manage my health”) (β =  − 0.219, p = 0.001), scale 5 (“appraisal of health information”) (β =  − 0.227, p < 0.001), scale 6 (“ability to actively engage with healthcare providers”) (β = − 0.135, p = 0.041) and scale 9 (“understanding health information well enough to know what to do”) (β =  − 0.166, p = 0.009) (Table 3). Patients with IBD only had significantly poorer scores in scale 3 (“actively managing my health”) (β =  − 0.196, p = 0.020) and scale 4 (“social support for health”) (β =  − 0.323, p < 0.001) compared to patients with cancer after adjusting for age, gender and education (Table 4).

**Table 2:** Multivariable linear regression analyses of each subscale of the HLQ comparing patients with liver cirrhosis and cancer.

	β	p-value
**Scale 1: Feeling understood and supported by healthcare providers**		
Liver cirrhosis (Reference: Cancer)	− 0.168	**0.005**
Gender	0.104	0.077
Age	0.045	0.433
University degree	0.143	**0.016**
**Scale 2: Having sufficient information to manage my health**		
Liver cirrhosis (Reference: Cancer)	− 0.227	** < 0.001**
Gender	0.112	0.054
Age	0.139	**0.016**
University degree	0.015	0.803
**Scale 3: Actively managing my health**		
Liver cirrhosis (Reference: Cancer)	− 0.263	** < 0.001**
Gender	0.098	0.089
Age	0.092	0.105
University degree	0.097	0.095
**Scale 4: Social support for health**		
Liver cirrhosis (Reference: Cancer)	− 0.253	** < 0.001**
Gender	0.157	**0.007**
Age	− 0.013	0.825
University degree	0.058	0.321
**Scale 5: Appraisal of health information**		
Liver cirrhosis (Reference: Cancer)	− 0.229	** < 0.001**
Gender	0.080	0.161
Age	0.043	0.442
University degree	0.186	**0.001**
**Scale 6: Ability to actively engage with healthcare providers**		
Liver cirrhosis (Reference: Cancer)	− 0.222	** < 0.001**
Gender	0.095	0.102
Age	0.044	0.435
University degree	0.147	**0.012**
**Scale 7: Navigating the healthcare system**		
Liver cirrhosis (Reference: Cancer)	− 0.154	**0.010**
Gender	0.093	0.118
Age	0.008	0.898
University degree	0.102	0.088
**Scale 8: Ability to find good health information**		
Liver cirrhosis (Reference: Cancer)	− 0.101	0.089
Gender	0.063	0.287
Age	− 0.065	0.262
University degree	0.191	**0.001**
**Scale 9: Understanding health information well enough to know what to do**		
Liver cirrhosis (Reference: Cancer)	− 0.189	**0.002**
Gender	0.167	**0.004**
Age	− 0.024	0.675
University degree	0.239	** < 0.001**

Significant values are in [bold].

Cancer was coded as 1 and liver cirrhosis was coded as 2.

Gender: men were coded as 1 and women as 2.

University degree was coded as 1 and no university degree was coded as 0.

**Table 3:** Multivariable linear regression analyses of each subscale of the HLQ comparing patients with liver cirrhosis and inflammatory bowel disease (IBD).

	β	p-value
**Scale 1: Feeling understood and supported by healthcare providers**		
Liver cirrhosis (Reference: IBD)	− 0.160	**0.014**
Gender	0.083	0.172
Age	0.039	0.545
University degree	0.121	**0.048**
**Scale 2: Having sufficient information to manage my health**		
Liver cirrhosis (Reference: IBD)	− 0.219	**0.001**
Gender	0.115	0.059
Age	0.141	**0.029**
University degree	0.011	0.862
**Scale 3: Actively managing my health**		
Liver cirrhosis (Reference: IBD)	− 0.098	0.128
Gender	0.158	**0.009**
Age	0.082	0.201
University degree	0.167	**0.006**
**Scale 4: Social support for health**		
Liver cirrhosis (Reference: IBD)	− 0.027	0.680
Gender	0.176	**0.004**
Age	0.035	0.588
University degree	− 0.039	0.524
**Scale 5: Appraisal of health information**		
Liver cirrhosis (Reference: IBD)	− 0.227	** < 0.001**
Gender	0.090	0.121
Age	− 0.064	0.298
University degree	0.219	** < 0.001**
**Scale 6: Ability to actively engage with healthcare providers**		
Liver cirrhosis (Reference: IBD)	− 0.135	**0.041**
Gender	0.081	0.189
Age	0.049	0.455
University degree	0.055	0.372
**Scale 7: Navigating the healthcare system**		
Liver cirrhosis (Reference: IBD)	− 0.087	0.187
Gender	0.099	0.112
Age	0.037	0.568
University degree	0.004	0.954
**Scale 8: Ability to find good health information**		
Liver cirrhosis (Reference: IBD)	− 0.074	0.258
Gender	0.030	0.625
Age	− 0.078	0.230
University degree	0.118	0.054
**Scale 9: Understanding health information well enough to know what to do**		
Liver cirrhosis (Reference: IBD)	− 0.166	**0.009**
Gender	0.130	**0.031**
Age	− 0.036	0.569
University degree	0.170	**0.005**

Significant values are in [bold].

IBD was coded as 1 and liver cirrhosis was coded as 2.

Gender: men were coded as 1 and women as 2.

University degree was coded as 1 and no university degree was coded as 0.

**Table 4:** Multivariable linear regression analyses of each subscale of the HLQ comparing patients with cancer and inflammatory bowel disease (IBD).

Development cohort	β	p-value
**Scale 1: Feeling understood and supported by healthcare providers**		
IBD (Reference: Cancer)	0.002	0.983
Gender	− 0.004	0.957
Age	− 0.008	0.927
University degree	0.166	0.025
**Scale 2: Having sufficient information to manage my health**		
IBD (Reference: Cancer)	0.035	0.679
Gender	0.010	0.900
Age	0.185	**0.025**
University degree	0.072	0.327
**Scale 3: Actively managing my health**		
IBD (Reference: Cancer)	− 0.196	**0.020**
Gender	0.010	0.899
Age	− 0.017	0.834
University degree	0.098	0.180
**Scale 4: Social support for health**		
IBD (Reference: Cancer)	− 0.323	** < 0.001**
Gender	0.028	0.712
Age	− 0.131	0.102
University degree	− 0.023	0.749
**Scale 5: Appraisal of health information **		
IBD (Reference: Cancer)	0.082	0.314
Gender	− 0.079	0.291
Age	− 0.055	0.484
University degree	0.293	** < 0.001**
**Scale 6: Ability to actively engage with healthcare providers**		
IBD (Reference: Cancer)	− 0.090	0.285
Gender	− 0.029	0.710
Age	0.047	0.571
University degree	0.076	0.301
**Scale 7: Navigating the healthcare system**		
IBD (Reference: Cancer)	− 0.012	0.886
Gender	− 0.037	0.636
Age	0.090	0.276
University degree	0.126	0.089
**Scale 8: Ability to find good health information**		
IBD (Reference: Cancer)	0.040	0.624
Gender	− 0.098	0.198
Age	− 0.039	0.631
University degree	0.240	**0.001**
**Scale 9: Understanding health information well enough to know what to do**		
IBD (Reference: Cancer)	0.025	0.755
Gender	− 0.017	0.822
Age	− 0.088	0.262
University degree	0.315	** < 0.001**

Significant values are in [bold].

Cancer was coded as 1 and IBD was coded as 2.

Gender: men were coded as 1 and women as 2.

University degree was coded as 1 and no university degree was coded as 0.

### Subgroup analyses of health literacy in patients with liver cirrhosis

**Figure 2:**
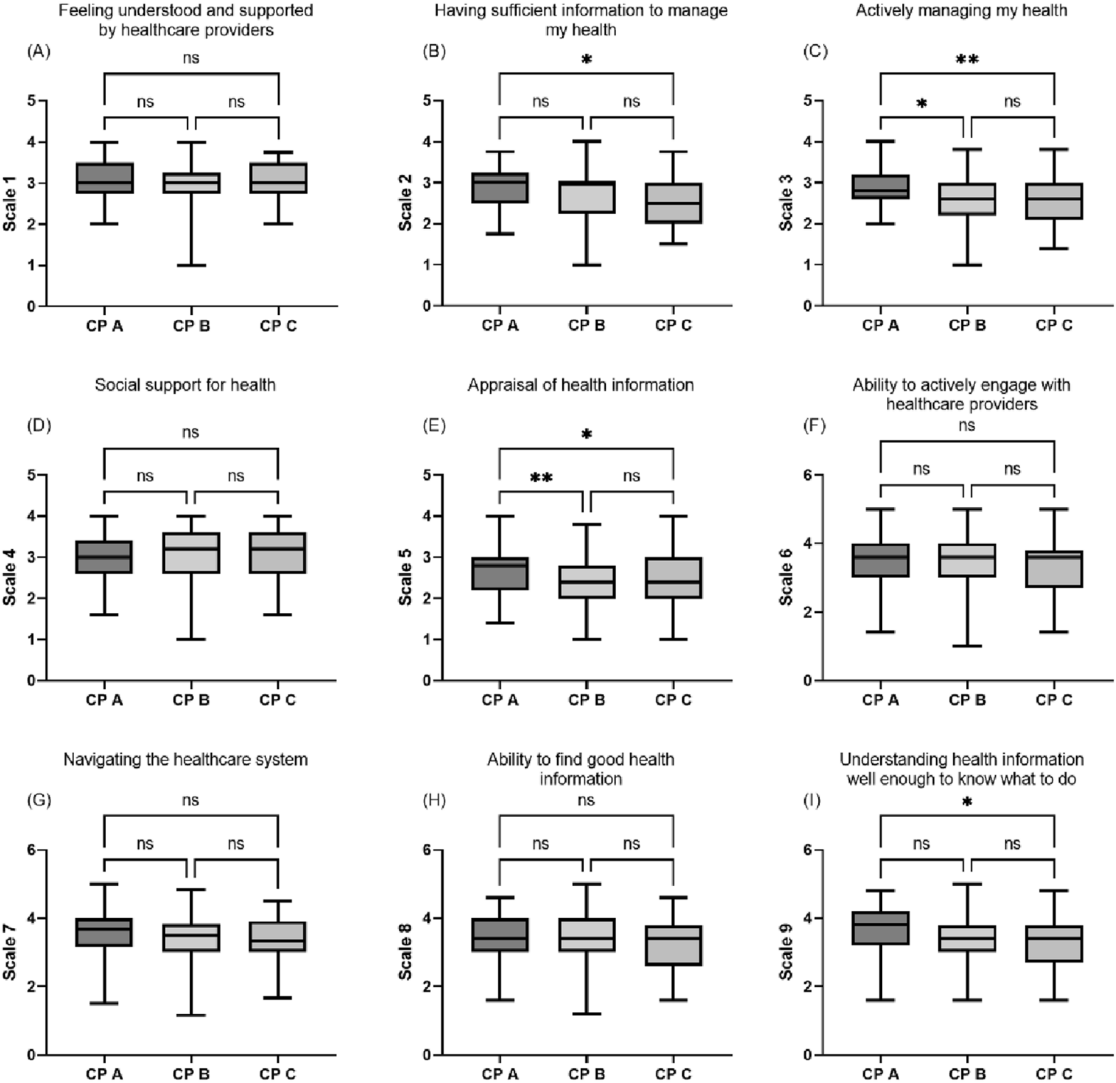
Comparison of the scores in the nine subscales of the HLQ between patients with liver cirrhosis Child–Pugh A, B and C. *p < 0.05, ** p < 0.01, ns = not significant. Comparisons of three groups were performed using an ANOVA followed by Tukey’s multiple comparison test.

To examine differences between disease stages in the cohort of patients with cirrhosis, we compared scores in the 9 subscales of the HLQ between patients with Child–Pugh A, B, and C cirrhosis. Here, patients with Child–Pugh C liver cirrhosis had poorer scores in the subscales scale 2 “having sufficient information to manage my health” (p < 0.05), scale 3 “actively managing my health” (p < 0.01), scale 5 “appraisal of health information” (p < 0.01) and scale 9 “understanding health information well enough to know what to do” (p < 0.01) compared to patients with Child–Pugh A liver cirrhosis (Fig. 2). The scores of patients with alcoholic liver cirrhosis only differed from patients with non-alcoholic liver cirrhosis in the subscales scale 2 “having sufficient information to manage my health” (p < 0.05), scale 3 “actively managing my health” (p < 0.01) and scale 5 “appraisal of health information” (p < 0.05) (supplementary Fig. 1).

## Discussion

In this study, we found that levels of health literacy differ significantly between patients with liver cirrhosis, IBD and gastrointestinal cancer. In particular, patients with liver cirrhosis scored lower in almost every subscale of the HLQ compared to patients with cancer. In addition, patients with cirrhosis had lower scores on several subscales of the HLQ, such as whether “having sufficient information to manage their health” or “understand health information well enough to know what to do”, compared to patients with IBD. These results were independent of age, gender, or higher educational attainment.

To the best of our knowledge, no previous study has examined differences in health literacy between patients with chronic gastrointestinal diseases. However, several studies investigated health literacy in patients with IBD or cancer alone. A study by Busch et al. investigated health literacy in patients with colorectal cancer and was able to demonstrate that patients with poorer health literacy were less likely to receive chemotherapy^20^. Another study on patients with cancer demonstrated that about 14% of the patients had low health literacy, which was associated with a longer hospital stay after cancer surgery^15^. Additionally, health literacy influences the timely diagnosis of symptomatic cancer^16,21^. In patients with IBD, evidence suggests that poorer health literacy is associated with worse patient-reported outcomes and depressive symptoms^18^. Another study indicated that the overall prevalence of poor health literacy in patients with IBD may be up to 24%^22^. Our study is not easily comparable to the aforementioned studies due to the variability in the applied tests to measure health literacy. Most of the previous studies only used rapid screening tests for poorer health literacy, which do not provide a fine granular picture of the different dimensions of health literacy, or only some highly selected dimensions of the HLQ^21^. Our study expands the existing literature by providing a robust head-to-head comparison of the whole spectrum of all dimensions defining health literacy between three frequent chronic gastrointestinal diseases, using the validated HLQ. All in all, health literacy in patients with cancer as well as IBD seems to be more or less comparable, while patients with IBD had slightly poorer results in terms of “actively managing my health” and “social support for health”.

Regarding health literacy in patients with liver cirrhosis, there is only limited evidence available. A study by Grydgaard et al.^17^ assessed the subscales “social support for health”, “ability to actively engage with healthcare providers” and “understand health information well enough to know what to do” of the HLQ in 105 outpatients with liver cirrhosis. While there was no relevant difference between Grydgaard’s and our current study regarding patients’ scores in the social support dimension, our German patients had remarkably lower scores in the “engagement and information dimension”^17^. However, in line with the study by Grydgaard et al., we did not observe a significant impact of an alcoholic etiology or disease stage on results in these three dimensions of the HLQ. The gaps between the results in both studies may most likely be explained by differences in healthcare systems.

In relation to previous studies, which focused mainly on one disease entity, our study expands the existing literature by demonstrating that patients with liver cirrhosis differ remarkably regarding their reported health literacy compared to patients with cancer in almost every subscale of the HLQ and also in five of nine subscales when compared to patients with IBD. Especially the comparably poor scores in the dimension “feeling understood and supported by healthcare providers” underscores the fact that liver cirrhosis may still be more stigmatizing than IBD or cancer. Potential explanations for the gaps in health literacy between the patient groups may be a larger online offering with detailed health information and more patient support groups for patients with IBD and especially patients living with cancer. However, it should also be acknowledged that educational differences between cohorts may have a large impact on health literacy, and this must be taken into account when interpreting our results.

When focusing on liver cirrhosis, our findings should be alarming for physicians treating those patients. It is a worrisome finding that patients with liver cirrhosis scored lowest in dimensions of health literacy such as “understanding health information well enough to know what to do”, “ability to actively engage with healthcare providers”, “having sufficient information to manage my health” or “appraisal of health information”. Additionally, these findings are aggravated by the fact that patients with poorer liver function, as reflected by the Child–Pugh score, even scored poorer in some of these dimensions. Interestingly, the association of an alcoholic etiology of liver cirrhosis and poorer results in subscales of the HLQ was only marginal or even non-existent, what is somehow counter-intuitive to daily life experiences of hepatologists treating patients with liver cirrhosis. Liver cirrhosis is a complex disease, and the management of complications requires a multidisciplinary approach with the involvement of patients and caregivers. In this context, our findings are worrisome as poorer health literacy is associated with a higher frequency of medication errors^3^. Keeping this in mind, our current findings seem to have several important implications for routine clinical practice and health care systems when dealing with patients with liver cirrhosis. Measures to improve health literacy in patients with liver cirrhosis seem to be urgent and may even result in a better prognosis of the disease. Given the fact that we did not observe any difference between the three patient groups in terms of the ability to find good health information, one starting point could be to improve the offer for e.g. patient information leaflets or online resources such as electronic health information, patient portals or even telemedicine options.

A strength of this study is its comparatively large sample size providing a comprehensive overview on health literacy in patients with liver cirrhosis, cancer and IBD. However, there are also some limitations that have to be acknowledged. Due to the cross-sectional design of the study, causality between the underlying diseases and poorer health literacy cannot be proven. Moreover, we are unable to prove whether improvements in health literacy may result in a better prognosis. Additionally, it is a limitation of our study that we did not investigate our patients with liver cirrhosis regarding the presence of minimal HE or alcohol toxic brain damage in detail. These factors could influence health literacy of patients with cirrhosis. Last, given the large number of tests used in our study to compare the different patient groups, the respective p-values have to be interpreted with caution. It has to be acknowledged that we present mostly pairwise group comparisons and no adjustments for multiple testing have been performed.

In conclusion, health literacy differs significantly among patients with chronic gastrointestinal diseases such as liver cirrhosis, IBD, or gastrointestinal cancer. Future studies are needed to identify further opportunities to improve health literacy, particularly in patients with cirrhosis.

## Methods

In total, 379 patients with either gastrointestinal cancer (just called “cancer” during the following manuscript) (n = 102), IBD (n = 86) or liver cirrhosis (n = 191) were recruited between January 2019 and April 2022 for this prospective, exploratory study at the Department of Internal Medicine I of the University Medical Center of the Johannes Gutenberg- University in Mainz, Germany. Detailed medical history was taken for each patient including social anamnesis and disease history.

All patients with cancer were consecutively recruited at the outpatient comprehensive cancer center that takes part of the Department of Internal Medicine I and were under treatment with systemic therapy. The underlying type of cancer was determined by biopsy and subsequent histology in all patients.

Patients with IBD were consecutively recruited at the outpatient department focused on patients with IBD that takes part of the Department of Internal Medicine I. All included patients suffered either from Crohn’s disease, ulcerative colitis or indeterminate colitis.

Patients with liver cirrhosis were consecutively recruited from the outpatient department of the Cirrhosis Centre Mainz (CCM) or from the regular wards of the Department of Internal Medicine I^23^. Patients with anamnestic active alcohol consumption were not approached. A subset of this cohort (n = 89) has been previously analyzed to identify predictors for poorer health literacy in patients with liver cirrhosis^23^. The leading etiology of underlying liver disease was determined according to clinical, serological and histological findings. Diagnosis of liver cirrhosis was made by histology, typical appearance in ultrasound or radiological imaging, endoscopic features of portal hypertension, and medical history. Model of end-stage liver disease (MELD) and Child–Pugh (CP) score were calculated to determine the severity of liver disease^23^. Inpatients were recruited at the end of their hospital stay after recompensation to avoid confounding effects of active infection or hepatic encephalopathy.

### Assessment of health literacy

As also previously described^23^, Health literacy was assessed using the Health Literacy Questionnaire (HLQ), which was validated for the German population in 2017^19,24^. The HLQ contains 44 items, which are divided into nine areas of health literacy^19^. The first five scales are scored on a 4-point Likert scale (ranging from strongly disagree to disagree, agree, and strongly agree), building part I. The other four scales, representing part II, are scored on a 5-point Likert scale where respondents are asked to rate the level of difficulty in undertaking a task (ranging from cannot do/ always difficult, usually difficult, sometime difficult, usually easy, and always easy). Higher scores indicate better health literacy.

The scales are subdivided into the following categories:

1. Feeling understood and supported by healthcare providers (HPS) (4 items),
2. Having sufficient information to manage my health (HSI) (4 items),
3. Actively managing my health (AMH) (5 items),
4. Social support for health (SS) (5 items),
5. Appraisal of health information (CA) (5 items),
6. Ability to actively engage with healthcare providers (AE) (5 items),
7. Navigating the healthcare system (NHS) (6 items),
8. Ability to find good health information (FHI) (5 items),
9. Understanding health information well enough to know what to do (UHI) (5 items).

It is important to note that every scale that purports to measure health literacy is its own scale and because scales have different items with different tasks/challenges, they can be easier or harder to answer (between scales). Consequently, one scale cannot be directly compared with another. Additionally, there are currently no evidence-based cut-off values available to dichotomize results.

Licence of the questionnaire was granted by the Swinburne University, Hawthorn, Australia. A trained healthcare professional assisted the patients to complete a paper version of the questionnaire.

### Ethics

The study was conducted in accordance with the ethical guidelines of the 1975 Declaration of Helsinki (6th revision, 2008). The study was approved by the ethics committee of the Landesärztekammer Rheinland-Pfalz (2019-14483). Written informed consent was obtained from all participants.

### Statistical analysis

Quantitative data are expressed as medians with interquartile ranges (IQR). Pairwise comparisons for quantitative variables were performed with the Mann–Whitney U Test. Comparison of three quantitative variables was performed using an ANOVA followed by Tukey’s multiple comparison test. Categorical variables are expressed as frequencies and percentages. For comparison of two or more patient groups, a chi-squared test was applied.

To exclude the potential bias in univariable analyses on health literacy caused by differences in age, gender distribution or education (university degree), we conducted various multivariable linear regression analysis including the respective gastrointestinal disease, age, gender and education (university degree).

Our complete data analysis is exploratory. Hence, no adjustments for multiple testing were performed. For all tests, we used a 0.05 level to define statistically significant deviations from the respective null hypothesis. However, due to the large number of tests, p-values should be interpreted with caution. Data were analysed using IBM SPSS Statistic Version 27.0 (Armonk, NY: IBM Corp.). Figures were drawn with GraphPad Prism Version 8.0.2 (GraphPad Software, California, US)^23^.

## Supplementary Information

Supplementary Information.

## Data Availability

The datasets generated during and/or analyzed during the current study are not publicly available but are available from the corresponding author on reasonable request.

## Abbreviations

AE: Ability to actively engage with healthcare providers
AL: Acute liver failure
AMH: Actively managing my health
CA: Appraisal of health information
CCM: Cirrhosis Centre Mainz
CP: Child–Pugh
FHI: Ability to find good health information
HLQ: Health Literacy Questionnaire
HPS: Feeling understood and supported by healthcare providers
HSI: Having sufficient information to manage my health
IQR: Interquartile ranges
MELD: Model of end-stage liver disease
NAFLD: Non-alcoholic fatty liver disease
NNHS: Navigating the healthcare system
SS: Social support for health
UHI: Understanding health information well enough to know what to do
