# Supplementary material for: Health literacy in gastrointestinal diseases: a comparative analysis between patients with liver cirrhosis, inflammatory bowel disease and gastrointestinal cancer
Source: Sci Rep. 2022 Dec 6;12:21072. doi: 10.1038/s41598-022-25699-w (PMC9726701; doi:10.1038/s41598-022-25699-w)
Supplement: Supplementary file 1 — Supplementary Information. [file 41598_2022_25699_MOESM1_ESM.pdf]

Supplementary table 1. Demographics and clinical characteristics of the patients with liver cirrhosis.

Variable		Patients with liver cirrhosis<br>n = 191
Age, y (IQR)		60 (52; 67)
Male gender, n (%)		117 (61.3)
University degree, n (%)		33 (17.3)
Aetiology	Alcohol, n (%)	115 (60.2)
	Viral hepatitis, n (%)	17 (8.9)
	NAFLD, n (%)	28 (14.7)
	Cholestatic/ Autoimmune, n (%)	6 (3.1)
	Other/mixed, n (%)	25 (13.1)
Median MELD score (IQR)		16 (11; 21)
Child-Pugh A/B/C, n (%)		47/95/49 (24.6 / 49.7 / 25.7)
History of ascites, n (%)		145 (75.9)
History of OHE, n (%)		57 (29.8)
Varices at study inclusion, n (%)		136 (71.2)
Albumin, g/l (IQR)		29 (24; 34)

Data are expressed as medians and interquartile ranges or as frequencies and percentages; NAFLD, non-alcoholic fatty liver disease; MELD, model for end-stage liver disease; OHE, overt hepatic encephalopathy.

Supplementary table 2. Demographics and clinical characteristics of the patients with cancer.

Variable	Patients with cancer n = 102
Age, y (IQR)	62 (55; 70)
Male gender, n (%)	80 (78.4)
University degree, n (%)	36 (35.3)
Cancer entity:	
Esophageal cancer, n (%)	28 (27.5)
Colon cancer, n (%)	8 (7.8)
Pancreatic cancer, n (%)	17 (16.7)
Cholangiocarcinoma, n (%)	13 (12.7)
Rectal cancer, n (%)	17 (16.7)
Gastric cancer, n (%)	9 (8.8)
Other, n (%)	10 (9.8)
Neoadjuvant chemotherapy, n (%)	19 (18.6)
Adjuvant/palliative chemotherapy, n (%)	83 (81.4)
ECOG performance status:	
0, n (%)	56 (54.9)
1, n (%)	38 (37.3)
2, n (%)	6 (5.9)
3, n (%)	2 (2.0)

Data are expressed as medians and interquartile ranges or as frequencies and percentages; ECOG, Eastern Cooperative Oncology Group.

Supplementary table 3. Demographics and clinical characteristics of the patients with inflammatory bowel disease.

**Variable**	**Patients with IBD**<br>**n = 86**
Age, y (IQR)	48 (33; 61)
Male gender, n (%)	42 (48.8)
University degree, n (%)	25 (29.1)
Ulcerative colitis, n (%)<br>Crohn's disease, n (%)<br>Indeterminate colitis, n (%)	41 (47.7)<br>42 (48.9)<br>4 (4.7)
Current medication:<br>5-ASA, n (%)<br>Adalimumab, n (%)<br>Ustekinumab, n (%)<br>Vedolizumab, n (%)<br>Golimumab, n (%)<br>Infliximab, n (%)<br>Tofacitinib, n (%)<br>Azathioprin, n (%)<br>Steroids, n (%)<br>Combination therapy, n (%)<br>No medication, n (%)	 8 (9.3)<br>6 (7.0)<br>11 (12.8)<br>13 (15.1)<br>2 (2.3)<br>5 (5.8)<br>1 (1.2)<br>2 (2.3)<br>1 (1.2)<br>33 (38.4)<br>4 (4.7)
Median time between diagnosis of IBD and study inclusion, months (IQR)	40 (66; 251)

Data are expressed as medians and interquartile ranges or as frequencies and percentages.

Supplementary figures

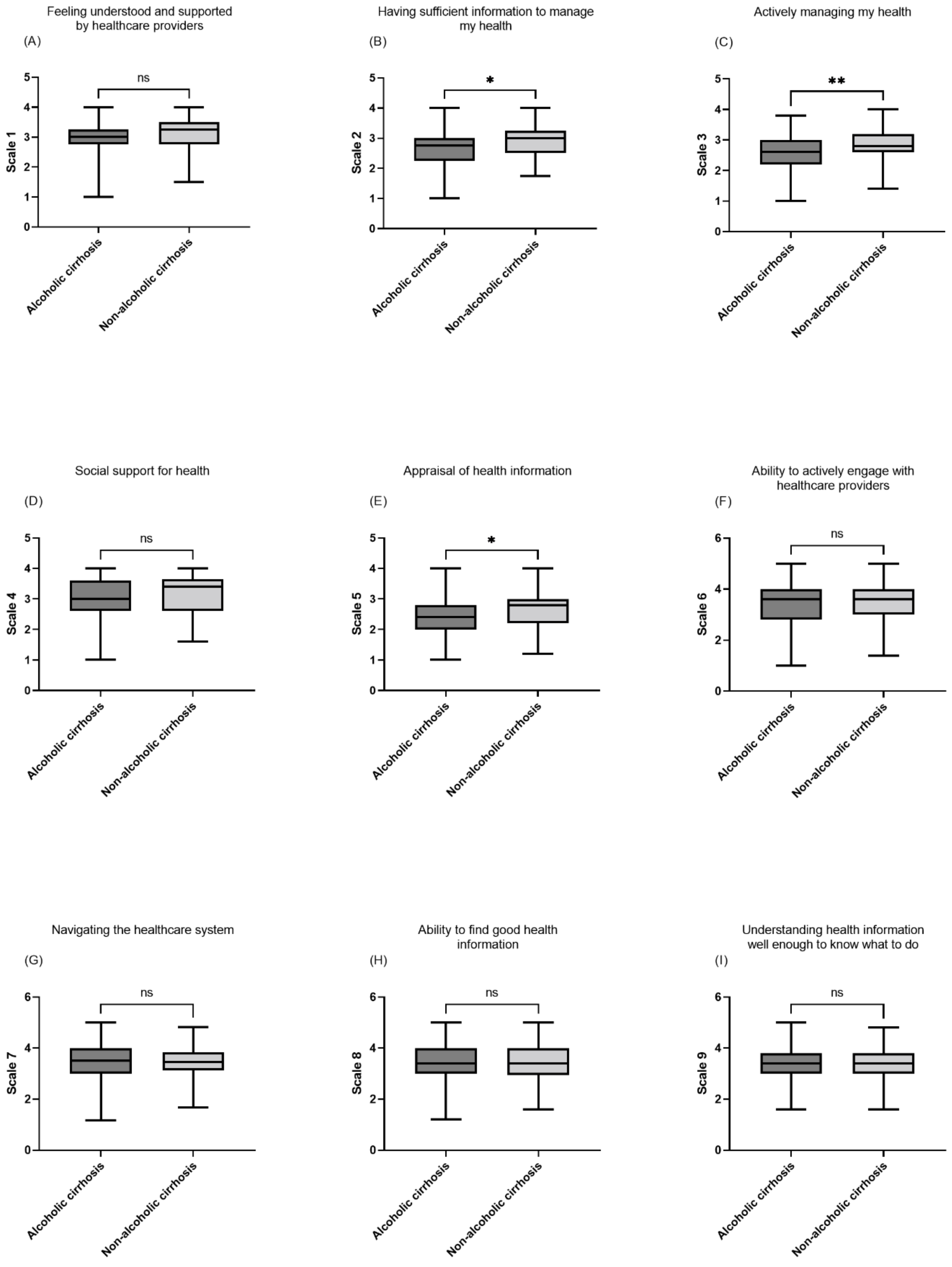

Supplementary figure 1. Comparison of the scores in the nine subscales of the HLQ between patients with alcoholic and non-alcoholic liver cirrhosis.

* p<0.05, ** p<0.01, ns=not significant
